# Effectiveness of Combination Therapy (Topical and Phototherapy) Versus Monotherapy (Phototherapy Alone) in the Treatment of Moderate to Severe Plaque Psoriasis: A Systematic Review and Meta-Analysis

**DOI:** 10.7759/cureus.117690

**Published:** 2026-10-09

**Authors:** Awadh Alamri, Ghala Alzhrani, Mustafa Sindi, Abdulrahman Alharbi, Abdulrahman Alsamadani, Rose Alraddadi, Saleh Aldraibi, Abdulaziz B Fathi, Hanan A Aljedani, Danah Aljahdali, Arwa Alharbi

**Affiliations:** 1 Dermatology, King Abdulaziz Medical City, Jeddah, SAU; 2 Medicine, King Saud bin Abdulaziz University for Health Sciences, Jeddah, SAU; 3 Dermatology, King Fahad General Hospital, Jeddah, SAU; 4 Dermatology, King Fahad Armed Forces Hospital, Jeddah, SAU; 5 Plastic and Reconstructive Surgery, King Abdulaziz Medical City, Jeddah, SAU; 6 Medicine and Surgery, King Abdullah International Medical Research Center, Jeddah, SAU; 7 Medicine, King Saud Bin Abdulaziz University for Health Sciences, Jeddah, SAU

**Keywords:** calcipotriol, combination therapy, narrowband uvb, phototherapy, plaque psoriasis, psoriasis, puva therapy, topical treatment, vitamin d analogs

## Abstract

Phototherapy remains an important treatment option for moderate-to-severe plaque psoriasis, but monotherapy may be limited by incomplete response, repeated treatment sessions, and cumulative ultraviolet exposure. This systematic review and meta-analysis evaluated the effectiveness and safety of topical therapy combined with phototherapy versus phototherapy alone. The review followed the Preferred Reporting Items for Systematic Reviews and Meta-Analyses (PRISMA) 2020 guidelines and included randomized controlled trials identified through database searches up to June 2025. Fifteen randomized clinical trials (RCTs) met the eligibility criteria. For Psoriasis Area and Severity Index (PASI) improvement, three studies provided comparable continuous data, and the pooled effect was not statistically significant (standardized mean difference or SMD=0.26; 95% CI, -0.31 to 0.84; p=0.37; I² =51%). Six studies contributed withdrawal data, with no significant difference between groups (Odds ratio or OR=0.98; 95% CI, 0.32 to 2.98; p=0.97; I² = 0%). Three studies contributed local skin adverse-event data, which favored monotherapy (OR=2.35; 95% CI, 1.05 to 5.26; p=0.04; I² = 38%). Individual trials frequently reported faster improvement, fewer treatment sessions, or lower cumulative ultraviolet exposure with combination therapy, but these findings were not uniform. Overall, combination therapy may improve treatment efficiency in selected patients, although local skin reactions may be more frequent and the evidence is limited by small studies, heterogeneous regimens, and short follow-up.

## Introduction and background

Plaque psoriasis is an immune-mediated inflammatory skin disease of chronic nature, which is marked by erythematous, scaly plaques that often develop on the scalp, trunk and extensor surfaces of the limbs. It affects about 2-3% of the world's population and has a considerable impact on the physical, psychological, and social health of the patients [1]. The pathology is based upon complicated genetic and immunologic mechanisms that lead to the hyperproliferation of keratinocytes, the activation of T-cells, and the dysregulation of cytokines [2]. Phototherapy has still been a mainstay in the treatment of moderate to severe psoriasis, as it helps lessen the scale, erythema, and lesion thickness by inhibiting immune responses in the skin [3]. In this study, we included narrowband ultraviolet B (NB-UVB), broadband UVB (BB-UVB), and psoralen plus ultraviolet A (PUVA) therapy. However, phototherapy as monotherapy is often limited by suboptimal response rates, excessive cumulative ultraviolet exposure, and high rates of recurrence following treatment discontinuation [3].

To improve treatment efficacy while limiting cumulative ultraviolet exposure, topical agents have increasingly been used as adjuncts to phototherapy. Commonly studied agents include corticosteroids, vitamin D analogues (e.g., calcipotriol and tacalcitol), and topical retinoids such as tazarotene and bexarotene. This combination is intended to enhance therapeutic response through complementary mechanisms while potentially reducing the total ultraviolet dose required to achieve disease control [4]. Topical therapies primarily target keratinocyte proliferation and local inflammation, whereas phototherapy exerts immunomodulatory and antiproliferative effects. Their complementary mechanisms provide the biological rationale for combination treatment. Besides, this combination can decrease the overall UV dose to emit a therapeutic effect, decreasing long-term chances of photoaging and photocarcinogenesis [5]. Current international treatment guidelines, including the joint American Academy of Dermatology-National Psoriasis Foundation guideline on phototherapy, continue to endorse combining phototherapy with topical adjuncts, such as vitamin D analogues, to enhance treatment response while limiting cumulative ultraviolet exposure [6].

Morison et al. compared PUVA alone with PUVA plus adjunctive corticosteroids in patients with plaque psoriasis; although this combination produced more rapid clearance, it was associated with a significantly higher recurrence rate during early follow-up compared with PUVA alone [7]. Later trials by Ramsay et al. and Woo and McKenna showed that calcipotriol with UVB or NB-UVB reduced the number of UV exposures and accelerated improvement without affecting final Psoriasis Area and Severity Index (PASI) scores [8,9]. Similarly, studies by Tzaneva et al. and Takahashi et al. confirmed that adjunctive topical therapies shortened treatment duration, reduced cumulative UV exposure, and improved clinical outcomes without increasing significant adverse effects [10,11]. Despite multiple controlled trials supporting adjunctive topical therapy with phototherapy, important evidence gaps remain due to small sample sizes, short follow-up periods, and methodological variability (e.g., differences in randomization procedures, blinding, and deviations from intended interventions) [7-11]. Differences in phototherapy modalities and topical agents also limit reliable comparison of efficacy and safety outcomes. Although several randomized controlled trials (RCTs) have evaluated topical agents combined with phototherapy, evidence directly synthesizing these studies to compare combination therapy with phototherapy alone remains limited.

This systematic review and meta-analysis aimed to compare the effectiveness and safety of combination therapy (topical agents plus phototherapy) versus phototherapy alone in patients with moderate-to-severe plaque psoriasis, focusing on treatment efficacy, safety, and tolerability to better inform evidence-based clinical practice.

## Review

Methods

Study Design and Protocol

This systematic review and meta-analysis was conducted in accordance with the Preferred Reporting Items for Systematic Reviews and Meta-Analyses (PRISMA) 2020 statement [12]. The protocol was prospectively registered in PROSPERO (CRD420251045401). The protocol defined the objectives, eligibility criteria, information sources, search strategy, and statistical methods in advance. Only RCTs were eligible for inclusion.

Eligibility Criteria

Eligible studies were RCTs comparing combination therapy (topical agents plus phototherapy) with phototherapy alone in patients of any age or gender with clinically diagnosed plaque psoriasis. Interventions included topical treatments such as calcipotriol, tazarotene, tacalcitol, or corticosteroids combined with NB-UVB, BB-UVB, or PUVA, while comparators used identical phototherapy parameters without topical agents. Outcomes included disease severity measures, including the PASI and PASI 50/75/90 responses (i.e., the proportion of patients achieving a ≥50%, ≥75%, or ≥90% reduction in PASI score from baseline) [13], or Physician’s Global Assessment (PGA) [14], with secondary outcomes assessing adverse events, treatment discontinuation, and quality of life. Exclusion criteria included the use of systemic agents, such as methotrexate or biologics; non-randomized studies; psoriasis types other than plaque psoriasis, such as guttate, pustular, or erythrodermic psoriasis; and incomplete or duplicate data.

Information Sources and Search Strategy

The relevant literature was identified by conducting a comprehensive search in PubMed/MEDLINE, Cochrane Central Register of Controlled Trials (CENTRAL), Embase, ScienceDirect, Scopus, Web of Science, and ClinicalTrials.gov. The database search began at the inception of each database through June 2025 to include all available trials. Manual searches of reference lists of included studies and previous reviews were also conducted in order to improve the coverage. Only publications written in English were added to ensure consistency in data extraction and quality evaluation.

The search strategy utilized both Medical Subject Headings (MeSH) terms and free-text keywords in the form of Boolean logic. The search included terms related to psoriasis, phototherapy, topical interventions, and randomized controlled trials. The complete search terms used for each database are provided in the appendix. Human study and clinical trial filters were applied where appropriate.

Study Selection and Data Extraction

All retrieved records were imported into Rayyan for study management and duplicate screening [15]. Two reviewers independently screened records in two stages: title/abstract screening followed by full-text eligibility assessment. Disagreements were resolved by consensus or, when necessary, by adjudication by a senior reviewer. The study selection process was documented in the PRISMA flow diagram (Figure 1).

**Figure 1:**
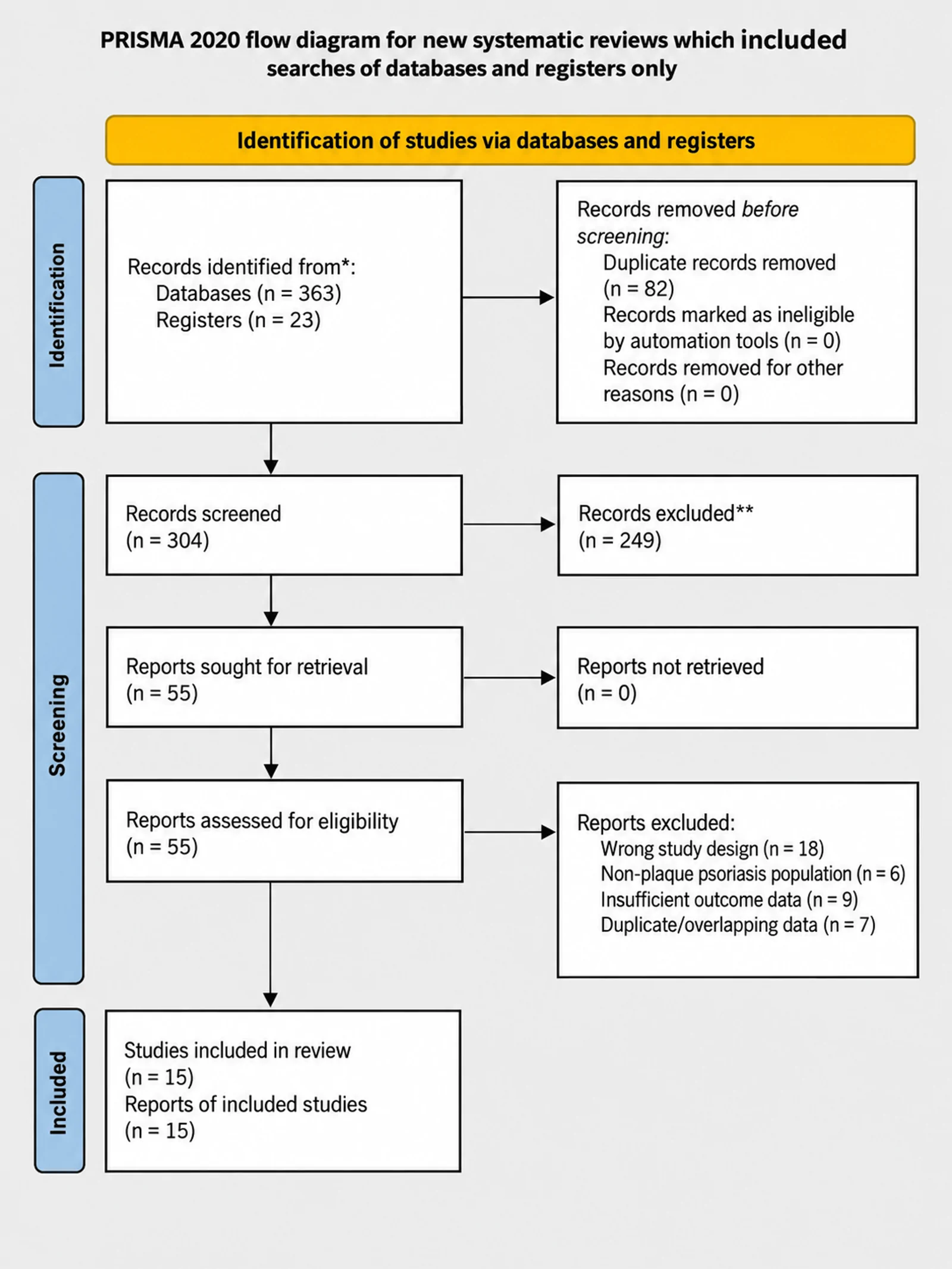
PRISMA 2020 flow diagram PRISMA: Preferred Reporting Items for Systematic reviews and Meta-Analyses. The search identified 386 records: 363 from bibliographic databases and 23 from ClinicalTrials.gov. After removing 82 duplicates, 304 records were screened; 249 were excluded, 55 reports were assessed for eligibility, and 40 were excluded with reasons, leaving 15 RCTs included in the systematic review.

Two reviewers independently extracted data using a standardized form, including study characteristics (author, year, country, design, sample size, and duration), participant characteristics, intervention and comparator details, and efficacy and safety outcomes. Disagreements were resolved by discussion or senior adjudication. When dispersion measures required derivation, calculations followed the Cochrane Handbook for Systematic Reviews of Interventions [16]. Extracted data were entered into Microsoft Excel (Microsoft Corp., Redmond, WA, USA) and verified before analysis in Review Manager (RevMan) 5.4.1 [17].

Synthesis of Data and Statistical Analysis

Meta-analyses were performed using Review Manager (RevMan) 5.4.1 [17]. Continuous outcomes were summarized using standardized mean differences (SMDs) with 95% confidence intervals (CIs), and dichotomous outcomes using odds ratios (ORs) with 95% CIs. A random-effects model was used. Statistical heterogeneity was assessed using the I² statistic and the Chi-square test. Subgroup analyses were not performed because the number of eligible studies within clinically comparable outcome groups was insufficient for reliable subgroup estimates.

Statistical significance was set at p<0.05. Forest plots were generated for PASI improvement, withdrawals due to adverse events, and local skin adverse events. Because outcome reporting differed substantially among the 15 included RCTs, each meta-analysis included only studies that reported sufficiently comparable, extractable data for that specific outcome.

Evaluation of Methodological Quality and Risk of Bias

Risk of bias was assessed using the Cochrane Risk of Bias 2 (RoB 2) tool for randomized trials [18]. The assessment considered bias arising from the randomization process, deviations from intended interventions, missing outcome data, measurement of the outcome, and selection of the reported result. Domain-level and overall judgments for all included RCTs are also presented.

Results

Study Selection

The database search identified 386 records (Ovid Journals=8, Directory of Open Access Journals (DOAJ)=7, MEDLINE=237, Cochrane Library=111, and ClinicalTrials.gov=23). After 82 duplicate records were removed, 304 unique records underwent title and abstract screening. Of these, 249 were excluded as reviews, non-randomized studies, or irrelevant reports, leaving 55 full-text articles for eligibility assessment. Forty full-text articles were excluded because of wrong study design (n=18), non-plaque psoriasis populations (n=6), insufficient outcome data (n=9), or duplicate/overlapping data (n=7). Fifteen RCTs were included in the systematic review. The complete selection process is shown in Figure 1.

Characteristics of Included Studies

Table 1 summarizes the characteristics of the 15 RCTs [7-11,19-28].

**Table 1:** Characteristics of randomized controlled trials comparing combination therapy (topical therapy plus phototherapy) with phototherapy alone for plaque psoriasis Prior-treatment and washout details for each study are provided in the appendix. 8-MOP: 8-Methoxypsoralen, AE: Adverse Event, BB-UVB: Broadband Ultraviolet B, BSA: Body Surface Area, F: Female, M: Male, max: maximum, min: minute, mo: month/months, Mono: Monotherapy, n: number, NB-UVB: Narrowband Ultraviolet B, NR: Not Reported, PASI: Psoriasis Area and Severity Index, PUVA: Psoralen plus Ultraviolet A, RCT: Randomized Controlled Trial, SD: Standard Deviation, TNF-α: Tumor Necrosis Factor-alpha, UV: Ultraviolet, UVA: Ultraviolet A, UVB: Ultraviolet B.

First author (Year)	Study design	Sample size	Follow-up eriod (months)	Age (mean/median ± SD or range, years)	Gender (M / F, n)	Psoriasis	Monotherapy	Combination therapy	
Takahashi et al., 2013 [11]	RCT, parallel group in Japan	40 total (4 groups; n = 10 each): calcipotriol alone; NB-UVB alone; calcipotriol + NB-UVB once weekly; calcipotriol + NB-UVB more than twice weekly.	12-week treatment period	Mono: 53.30 ± 4.42 Combo: 53.50 ± 4.47	Mono: 8/2 Combo: 9/1	Plaque-type psoriasis with PASI >10, skin phototypes III and IV.	Narrowband UVB (NB-UVB) (TL01) + Heparinoid emollient wavelength: 311-312 nm 3 times weekly	Calcipotriol 50 µg/g Ointment + NB-UVB; NB-UVB: Identical to monotherapy Calcipotriol 50 µg/g; Ointment: Twice daily (with a 2-hour gap before UVB irradiation)	
Özkan et al., 2012 [19]	RCT in Turkey	30 • Monotherapy (UVB alone - Group 1): 10 • Combination (UVB + Psoralen - Group 2): 10 • Combination (UVB + Calcipotriol - Group 3): 10	10 weeks	29.1 ± 11.2 years (Range: 18-53)	16/14	Stable, localized, chronic plaque-type psoriasis	Broadband UVB 3 times per week	0.1% Psoralen Gel (Group 2), Calcipotriol Ointment (Group 3), and BB-UVB UVB: Identical to monotherapy 3 times per week	
Ehsani et al., 2011 [20]	RCT Left-Right Comparison Trial in Iran	10 patients (20 lesions analyzed) Combination (8-MOP + NB-UVB): 10 lesions Monotherapy (Placebo + NB-UVB): 10 lesions	13 weeks	36.50 ± 12.85 years (Range: 25-65)	4/6	Stable plaque-type psoriasis (<20% BSA), with symmetric lesions on the shins.	NB-UVB (wavelength: 311-313 nm 3 times per week	8-Methoxypsoralen (8-MOP) 0.001% Cream+UVB NB-UVB: Identical to monotherapy 3 times per week	
Magliocco et al., 2006 [21]	RCT double-blinded in the USA	9	10 weeks (Topical therapy). 8 weeks (NB-UVB).	Range: 31-77 years	7/2	Psoriasis: moderate to severe Affects 3% or greater of the body's surface area	NB-UVB + Placebo More than twice a week	Bexarotene 1% Gel +NB-UVB NB-UVB: Identical to monotherapy Bexarotene: 3 times weekly Started every other day (Week 1), then once daily (Week 2), then twice daily (as tolerated).	
Grundmann-Kollmann et al., 2004 [22]	RCT in Germany	30 Combination (NB-UVB + cream PUVA): 10 Monotherapy 1 (NB-UVB alone): 10 Monotherapy 2 (Cream PUVA alone): 10	24 weeks (6 months) post-treatment	Range: 22-73 years	19/11	Moderate to severe chronic plaque-stage psoriasis	NB-UVB (whole-body) UVA for cream PUVA (selected plaques) wavelength NB-UVB: 311 nm; UVA: 320-400 nm (peak 365 nm) 4 times per week	8-Methoxypsoralen (8-MOP) 0.001% Cream+UVB UVB: Identical to monotherapy 4 times per week	
Woo and McKenna, 2003 [9]	RCT, parallel group in the UK	50 (36 completed) Combo: 25 Mono: 25	Up to 20 treatment sessions, plus 10-week follow-up	Combo: 38.2 years Mono: 43.3 years	Combo: 12/13 Mono: 16/9	Chronic plaque and/or guttate	Narrowband UVB (NB-UVB) (TL01)+ Placebo emollient wavelength: 311-312 nm for 3 times weekly	Calcipotriol 50 µg/g Ointment+NB-UVB NB-UVB: Identical to monotherapy Calcipotriol 50 µg/g Ointment: Twice daily (after phototherapy)	
Rim et al., 2002 [23]	RCT outpatient study in South Korea	28 Combination (Calcipotriol+UVB): 10 Monotherapy (UVB alone): 18	43 days	Combination: 39.7 ± 12.5 • Monotherapy: 39.7 ± 14.8	• Combination: 3/7 • Monotherapy: 7/11	Chronic plaque psoriasis affecting >5% of body surface area	Narrowband UVB (NB-UVB) (TL01) wavelength: 311-312 nm for 3 times weekly	Calcipotriol 50 µg/g Cream+NB-UVB NB-UVB: Identical to monotherapy Calcipotriol 50 µg/g Cream: Twice daily (with a 2-hour gap before UVB irradiation)	
Tzaneva et al., 2002 [10]	RCT Observer-blinded, intrapatient comparison study in Austria	24	Treatment: up to 6 weeks Follow-up: 18 months	Median: 49 years	16/8	Skin types III/IV: 30% Skin types I/II: 15% or 5%. (no erythema) or 10% (barely perceptible erythema).	PUVA Monotherapy Psoralen: Oral 8-MOP (0.6 mg/kg) 1 hour before UVA. UVA: 4 times/week. Started at 70% of Minimal Phototoxic Dose (MPD). Dose increased by 15-30% per session based on skin type and erythema.	1- Tacalcitol + PUVA PUVA: Identical to monotherapy Topical: Tacalcitol ointment applied once daily to designated plaques. 2- Tazarotene + PUVA PUVA: Identical to monotherapy above. Topical: 0.1% Tazarotene gel applied once daily. If irritated, switched to 0.05% gel.	
Ring et al., 2001 [24]	RCT, double-blinded, parallel group in Germany	102 Calcitriol+UVB: 49 Vehicle+UVB: 53	8-week treatment phase	Mean: 44.5 years (Range: 22-74)	NR	chronic plaque-type psoriasis, global severity ≥ grade 2	BB-UVB wavelength: 290–320 nm Up to 24 sessions over 8 weeks	Calcipotriol 3 µg/g Ointment+BB-UVB BB-UVB: Identical to monotherapy Calcipotriol 3 µg/g Ointment: Twice daily (morning and evening)	
Behrens et al., 2000 [25]	RCT, Half-side, comparative study in Germany	10	4-week treatment period	NR	NR	stable plaque-type psoriasis.	Narrowband UVB (NB-UVB) + Emollient: for 5 times weekly wavelength: 311 nm	Tazarotene 0.05% Gel +NB-UVB NB-UVB: Identical to monotherapy Tazarotene: 5 times weekly Thin film once daily (every evening)	
Koo et al., 2000 [26]	RCT outpatient study in the USA	54 patients (108 plaques analyzed) Combination (Tazarotene + UVB): 36 plaques Monotherapy (UVB alone): 36 plaques	81 days	mean 41.9 (13.5) Range (23-77)	49/5	stable plaque psoriasis on ≤50% body surface area with two bilaterally symmetric target plaques	Narrowband UVB (NB-UVB): for 3 times weekly	Tazarotene 0.1% Gel +NB-UVB NB-UVB: Identical to monotherapy Tazarotene: 3 times weekly	
Ramsay et al., 2000 [8]	RCT, single-blinded, parallel group, multicenter in Canada	159 Combo: 80 Mono: 79	12-week treatment phase, 12-week follow-up phase	Combo: 45.7 (14.3) Mono: 43.2 (13.5)	Combo: 53/31 Mono: 31/34	Combo: I/II: 35.7 III/IV: 64.3 Mono: I/II: 45.1 III/IV: 55.1	Broadband UVB (BB-UVB) 3 times weekly for 12 weeks 3 times weekly	Calcipotriol 50 µg/g Cream+BB-UVB BB-UVB: Identical to monotherapy Calcipotriol 50 µg/g Cream: Applied twice daily (max 100g/week)	
Speight and Farr, 1994 [27]	RCT observer-blinded, Within-patient, in the UK	11	6-week treatment period, 6-month follow-up	Mean: 50 years (Range: 23-71)	8/3	plaque-type psoriasis	PUVA Monotherapy+placebo Twice Weekly	Calcipotriol 50 µg/g +PUVB PUVB: Identical to monotherapy Calcipotriol 50 µg/g Ointment: Thin film, Twice daily	
Brands et al., 1999 [28]	RCT outpatient study in the Netherlands	53 Combination (UVB+Calcipotriol): 25 Monotherapy (UVB alone): 28	NR	Mean: 45 years (Range: 14-76)	NR	Plaque-type psoriasis; skin phototypes II-IV	Narrow-band UVB (TL-01) wavelength: 311-312 nm 3 times weekly	Calcipotriol 50 µg/g +NUVB NUVB: Identical to monotherapy Calcipotriol 50 µg/g Ointment: Thin film, Twice daily 3 times weekly	
Morison et al., 1978 [7]	RCT outpatient study in the USA	116 Group 1: 58 (PUVA + concurrent topical therapy: dithranol 20, steroids 19, tar 19); Group 2: 40 (topical 6 weeks pre-PUVA + PUVA: dithranol 19, steroids 19, tar 2); Control: 18 (PUVA only)	Clearance phase and 4-month maintenance/follow-up	Mean: 40 years	NR	Patients with psoriasis vulgaris 12% had active arthritis of the psoriatic type	PUVA wavelength: 320-400 nm twice weekly	Dithranol (0.25-1%), Fluocinolone acetonide (0.01-0.025%) (steroid), or Tar + PUVA Daily	

The studies were published between 1978 and 2013 and were conducted across North America, Europe, and Asia. Most enrolled adults with chronic plaque psoriasis and evaluated NB-UVB, BB-UVB, or PUVA in combination with topical agents, most commonly vitamin D analogues. Other adjunctive agents included tazarotene, bexarotene, topical psoralen preparations, corticosteroids, dithranol, and tar. Treatment schedules and follow-up durations varied across studies, contributing to clinical heterogeneity. Although studies involving psoriasis subtypes other than plaque-type were excluded a priori per the predefined eligibility criteria, some included trials enrolled patients with more localized or stable disease; this contributes to the population heterogeneity discussed later, and our overall conclusions have been tempered accordingly to avoid overstating the applicability of the findings to moderate-to-severe plaque psoriasis specifically. We also note that one included trial, Woo and McKenna (2003) [9], enrolled a population described in the original publication as having chronic plaque and/or guttate psoriasis rather than exclusively plaque-type disease; this was not identified during full-text eligibility screening, and although the majority of enrolled patients had chronic plaque psoriasis, we acknowledge this as an additional source of population heterogeneity, discussed further with respect to individual outcomes below. In addition, although the search was conducted through June 2025, no additional randomized controlled trials directly comparing combination therapy with phototherapy alone were identified after 2013 for this specific comparison; the absence of contemporary trials reflects a genuine gap in the recent literature on this comparison rather than a limitation of the search strategy and is an important consideration when interpreting the applicability of these findings to current clinical practice.

Efficacy and Safety Outcomes

Table 2 summarizes the efficacy and safety outcomes reported across the included RCTs.

**Table 2:** Clinical efficacy and safety outcomes of combination therapy versus phototherapy alone for plaque psoriasis AE: adverse event; BSA: body surface area; CI: confidence interval; Combo: combination therapy; 5-MOP: 5-methoxypsoralen; 8-MOP: 8-methoxypsoralen; LDH: lactate dehydrogenase; Mono: monotherapy; NB-UVB/NBUVB: narrowband ultraviolet B; NR: not reported; PASI: Psoriasis Area and Severity Index; PASI-50/PASI-75: at least 50%/75% improvement in PASI; PDI: Psoriasis Disability Index; PSI: Psoriasis Severity Index; PUVA: psoralen plus ultraviolet A; SD: standard deviation; UV: ultraviolet; UVB: ultraviolet B; UVA: ultraviolet A.

First author (Year)	Psoriasis severity	Improvement	Patient satisfaction	Withdrawals due to AEs (n/%)	Local skin reactions (n/%)	Systemic side effects (n/%)	Conclusion
Takahashi et al., 2013 [11]	Combo: most marked and rapid reduction in PASI and self-PASI scores The combo was significantly lower than the Mono at 12 weeks.	Combo: showed a rapid reduction in PASI scores	NR	0	0	0	The combination of Calcipotriol + frequent NB-UVB was significantly more effective than NB-UVB monotherapy with an emollient, resulting in a more marked and rapid reduction in PASI scores and a PASI-75 response rate of 70% at 12 weeks.
Özkan et al., 2012 [19]	% PASI Improvement: • Group 1 (UVB alone): 33.9% • Group 2 (UVB+Psoralen): 29.9% • Group 3 (UVB+Calcipotriol): 67.2% Mean change in PASI score Group 1: 2.72 to 1.80 Group 2: 3.25 to 2.28 Group 3: 3.66 to 1.20	Based on % PASI/PSI reduction. At week 10: • >50% lesion clearance: Group 1: 40%; Group 2: 10%; Group 3: 100%	NR	0	Erythema and pruritus	NR	Our findings suggest that combining calcipotriol with targeted phototherapy yields better results than using targeted microphototherapy alone or in combination with psoralen. Additionally, we discovered that incorporating a topical photosensitizer reduces the number of treatment sessions required.
Ehsani et al., 2011 [20]	Mean PASI improvement was 55% PSI Baseline: Combo: 6.30 ± 1.34; Mono: 6.30 ± 1.34 Final (13th session): Combo: 2.90 ± 0.88; Mono: 3.70 ± 0.68 % Decrease: Combo: 54.0%; Mono: 41.3%	Patient-level (Global): PASI 75: 0%; PASI 50: 90%	NR	0	Pigmentation was the only reported AE. No burning or blistering.	NR	A notable reduction in severity scores was noted in both groups, although none of the lesions were completely resolved by the conclusion of the study. The reduction observed in the 8MOP group was more pronounced than in the control group, but the difference did not reach statistical significance. The sole side effect reported was pigmentation, which appeared in all patients treated with 8MOP after two weeks.
Magliocco et al., 2006 [21]	PASI mean (95%CI) decrease in score from baseline Combo: 67.6% (50.9%-84.3%) Mono: 48.2% (24.0%-72.5%)	NR	NR	1 patient (10%) withdrew due to skin irritation from the gels.	Macular erythematous rash (n=2, 22%) Skin irritation (n=2, 22%)	Worsening/fluctuating hypertension (n=4), deemed unrelated to study drugs.	NBUVB treatment started two weeks after the introduction of topical therapy. The study faced limitations, including a small sample size and the use of interim analysis. According to scores for the target lesions, the combination of bexarotene gel 1% with NBUVB demonstrated significantly greater effectiveness than placebo combined with NBUVB.
Grundmann-Kollmann et al., 2004 [22]	100% of patients in all groups achieved clearance (PASI <4)	Combination: 3-4 weeks Monotherapy 1 (NB-UVB): 5-7 weeks Monotherapy 2 (Cream PUVA): 5-7 weeks	NR	0	Moderate erythema	NR	Our findings suggest that combining narrowband UVB with cream PUVA is considerably more effective than either treatment alone. Additionally, the total UV exposure was notably reduced with the combination therapy. Therefore, we conclude that cream PUVA is a viable adjunct to narrowband UVB, particularly for regions of the body that are slower to respond to treatment.
Woo and McKenna, 2003 [9]	Final PASI scores Combo: 1.3 Mono: 2.3	Significantly faster for Combo. Significant difference in PASI reduction in the first 8 sessions.	NR	Combo: 1 (4%), Mono: 1 (4%)	Combo: burning (n=6), Pruritus (n=2), Erythema (n=1). Mono: burning (n=2), Pruritus (n=1), Polymorphic light eruption (n=1).	NR	The approach of integrating narrowband TL01 phototherapy with topical calcipotriol offers a well-tolerated and effective strategy for treating psoriasis. While both treatment groups achieved comparable results in PASI and PDI scores, we observed a UVB-sparing effect, which may reduce the risk of skin cancer.
Rim et al., 2002 [23]	NR	90% (9/10) in combination vs 61.1% (11/18) in monotherapy achieved "Grade IV" clearance	NR	Combination: 1/10 (10%) due to severe itching. • Monotherapy: 1/18 (5.6%) due to burn with vesicle formation.	Itching and burning	NR	Other findings revealed no significant difference in the total UVB dose needed to reach grade IV between the two groups, though the calcipotriol–narrow-band UVB group had a slightly lower dose. However, a higher percentage of patients in the combination group achieved grade IV by treatment's end, and this method was more effective in reducing the Psoriasis Area and Severity Index early on.
Tzaneva et al., 2002 [10]	PSI Median (95%CI) Tacalcitol plus PUVA Baseline: 10 (8-11) At 6 weeks: 1 (0-2) Tazarotene plus PUVA Baseline: 10 (8-11) At 6 weeks: 1 (0-2) PUVA monotherapy Baseline: 10 (8-11) At 6 weeks: 1 (0-2)	Time: Significantly faster for combinations. Cumulative exposures to clearance: Combo: 14 vs. Mono: 16 (P<0.01).	NR	0 (No withdrawals due to AEs. 3 patients defaulted for reasons unrelated to treatment).	Tacalcitol: Mild irritant dermatitis (n=1), Hypertrichosis (n=1). Tazarotene (0.1%): Dryness (n=5), Irritant contact dermatitis (n=4), Itching (n=3), burning (n=1).	Severe nausea from oral 8-MOP in 3 patients, who were switched to 5-MOP.	Tacalcitol ointment and tazarotene gel demonstrate similar efficacy in improving outcomes of PUVA therapy in individuals with chronic plaque-type psoriasis. In addition to speeding up treatment response, both agents may help minimize the potential long-term risks of PUVA therapy by reducing UVA exposure.
Ring et al., 2001 [24]	Mean % PASI decrease: Combo 65% vs. Mono 43%	Considerable improvement/clearing: Combo 45% vs. Mono 21%. Significant difference from week 1 (P<0.05). Time: Significant improvement in global score from week 1.	Cosmetic acceptability rated 'good' by 80% (Combo) vs. 74% (Mono).	3 patients (1 from each group for rash; 1 from Combo for unrelated diverticulitis)	Skin-related AEs Combo: 6 Mono: 6	Transient, asymptomatic hypercalcaemia in 2 Combo patients.	The combination of calcitriol ointment at 3 mg/g with UVB phototherapy shows significant promise as a treatment for chronic plaque psoriasis. The study highlights the high efficacy and good local tolerance of this approach, suggesting that combining calcitriol with UVB therapy could be an advantageous long-term management strategy for patients with chronic plaque psoriasis.
Behrens et l., 2000 [25]	The median PASI score decreased after 4 weeks. Combo: 6.5 (95% CI, 5.29-7.91) Mono: 9.5 (95% CI, 7.70-11.70) 64% reduction (combination), 48% (UVB alone)	Significant improvement in PASI was noted at 2 weeks.	NR	0	Mild local cutaneous irritation (transient burning, erythema) with tazarotene.	NR	Both treatment approaches significantly reduced PASI scores, with the skin regions treated with narrow-band UVB phototherapy and topical tazarotene showing notably greater improvement. Combining tazarotene with narrow-band UVB phototherapy results in more rapid, more efficient resolution of psoriasis than UVB (311 nm) alone.
Koo et al., 2000 [26]	NR	At day 81: 82% of plaques in the combination group had ≥75% improvement vs. 50% in the monotherapy group. Median time to ≥75% improvement: 53 days (combination) vs. 81 days (monotherapy).	NR	2/54 patients (3.7%) due to AEs (unrelated to topical therapy)	Combination: skin irritation (5 plaques), burning sensation (1 plaque), pruritus (1 plaque)	NR	The combination of tazarotene with UVB phototherapy resulted in more rapid and greater reductions in plaque height and scaling during treatment. This approach produced at least a 50% improvement over baseline measurements prior to treatment, while requiring significantly lower total UVB exposure than the vehicle gel with UVB light or UVB therapy alone. Additionally, no atypical photosensitivity was reported in the group receiving tazarotene alongside UVB treatment.
Ramsay et al., 2000 [8]	PASI mean percentage reduction (SD) Combo: 77.0% (39.4) Mono: 80.1% (25.2)	Investigator's "Marked Improvement/Clearance": combo: 72.5%, Mono: 77.2% Time: Significantly faster for Combo. Median exposures to 80% PASI reduction: Combo 12 vs. Mono 19 (P<0.001). Median exposures to clearance: Combo 22 vs. Mono 25 (P<0.001).	Patient's "Marked Improvement/Clearance": Group A: 70.0%, Group B: 74.7%	combo: 1 (1.3%), mono: 4 (5.1%) The most common adverse events were UVB-related (burn, pruritus, and erythema)	Combo: at least one skin-related adverse event Mono: 43 (53.8%)	NR	This research indicates that applying calcipotriol cream in conjunction with broad-band UVB therapy twice a week is an effective and safe method for managing extensive psoriasis. When compared with the conventional treatment of broad-band UVB three times a week, this combination is equally effective and safe, offering the benefit of fewer UVB sessions and a lower energy density required to achieve an 80% reduction in PASI scores and clear the psoriasis.
Speight and Farr, 1994 [27]	Clinical Clearance: Calcipotriol plaque cleared first in 7/11 patients (64%).	Calcipotriol-treated plaques were consistently judged to be better or cleared first in 9/11 patients (82%). Significantly faster for Combo. Clearance occurred earlier with calcipotriol.	NR	1 patient due to poor compliance with topical treatment.	NR	NR	Combining topical agents with phototherapy is more effective than phototherapy alone for treating plaque psoriasis. This approach leads to faster clearance of psoriatic plaques and significantly reduces the cumulative dose of ultraviolet radiation required for treatment, thereby minimizing long-term risks.
Brands et al., 1999 [28]	PASI response (%) UVB+Calcipotriol: 79.3% reduction UVB alone: 75.5% reduction	NR	NR	UVB+Calcipotriol: 2/25 (8%) due to (peri)lesional burning and itching. UVB alone: 0/28.	UVB+Calcipotriol: 2 patients with (peri)lesional irritation; 3 patients complained that the ointment was too greasy.	NR	Our findings indicate that low-dose narrow-band UVB phototherapy is an effective psoriasis treatment. Additionally, we found that using calcipotriol ointment in combination with this therapy does not improve overall results. Consequently, we recommend against combining calcipotriol ointment with low-dose narrow-band UVB phototherapy, as the UVB treatment is highly effective on its own.
Morison et al., 1978 [7]	Clearance of Psoriasis to <1% Body Surface Area (BSA) (PUVA alone): 18/18 (100%) (PUVA + Dithranol): 19/20 (95%) (PUVA + Steroids): 19/19 (100%) (PUVA + Tar): 16/19 (84%)	Overall Mean for all patients: 17 PUVA treatments over 69 days to a final UVA dose of 15 J/cm².	Percentage of patients dissatisfied with treatment regimen: Dithranol: 72% Steroid: 63% Tar: 48%	NR	Dithranol caused skin irritation and staining.	Nausea reported. 5 patients had transient LDH elevation.	The findings of this research suggest that adjunctive topical treatments in combination with PUVA may be beneficial for managing scalp psoriasis. Furthermore, these therapies could be useful for stubborn localized areas. However, the three topical agents examined in this study do have certain drawbacks. Steroids seem to have a negative interaction with PUVA; dithranol carries the risk of causing chemical burns and triggering the Koebner phenomenon, while tar was found to be relatively ineffective.

Several trials reported faster clinical improvement, earlier lesion clearance, fewer treatment sessions, or lower cumulative ultraviolet exposure with combination therapy, although final efficacy was not consistently superior across studies. Both strategies were generally well tolerated. Local reactions, including erythema, burning, pruritus, and irritation, were reported more often with some combination regimens, particularly those containing topical retinoids, whereas systemic adverse events were uncommon.

Meta-analysis of Pooled Outcomes

PASI improvement: Three RCTs [8,21,25] provided sufficiently comparable continuous PASI data (means and standard deviations) for pooling. The pooled standardized mean difference favored combination therapy but was not statistically significant (SMD=0.26; 95% CI, -0.31 to 0.84; p=0.37). Heterogeneity was moderate (I² = 51%; p=0.13) (Figure 2).

**Figure 2:**
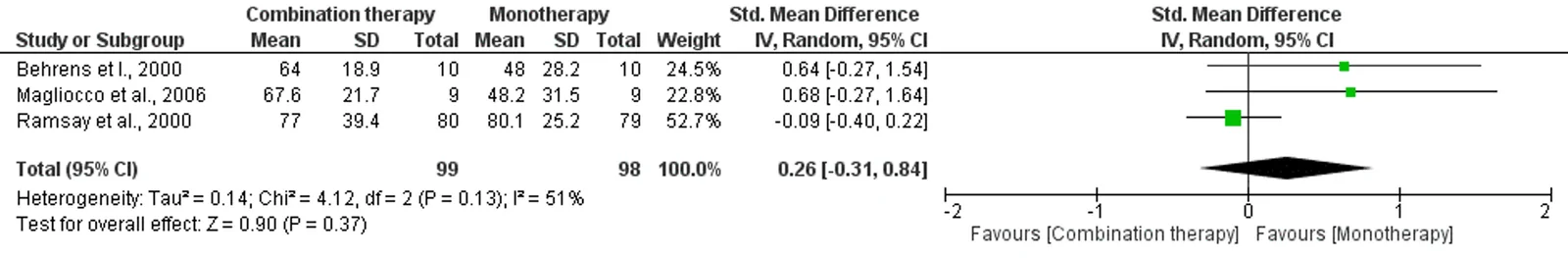
Forest plot of Psoriasis Area and Severity Index (PASI) improvement Three RCTs [8,21,25] were included because they reported sufficiently comparable continuous PASI data (means and standard deviations) for standardized mean-difference pooling. Green squares represent the effect estimate from each individual study; larger squares indicate greater study weight, and horizontal lines represent 95% confidence intervals. The black diamond represents the pooled effect estimate, with its width representing the pooled 95% confidence interval.

Withdrawals due to adverse events: Six RCTs [8,9,21,23,24,28] contributed data to the available quantitative analysis of withdrawals due to adverse events. The pooled analysis showed no significant difference between combination therapy and monotherapy (OR=0.98; 95% CI, 0.32 to 2.98; p=0.97), with no observed heterogeneity (I² = 0%; p=0.68) (Figure 3).

**Figure 3:**
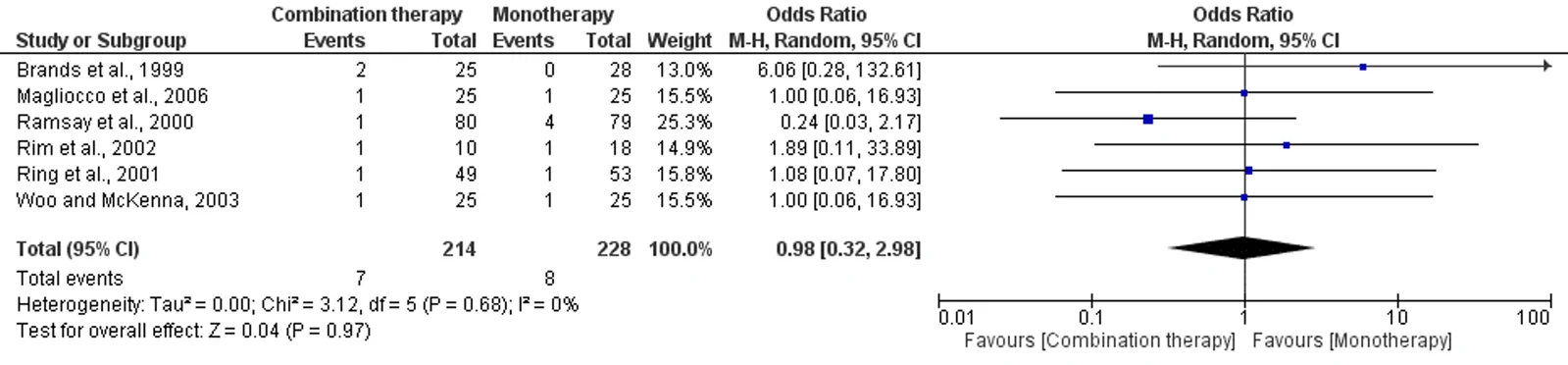
Forest plot of withdrawals due to adverse events Six RCTs [8,9,21,23,24,28] were included because they reported extractable withdrawal data for this outcome. Blue squares represent the effect estimate from each individual study; larger squares indicate greater study weight, and horizontal lines represent 95% confidence intervals. The black diamond represents the pooled effect estimate, with its width representing the pooled 95% confidence interval.

Because one of these six trials, Woo and McKenna [9], enrolled a population that included patients with guttate as well as chronic plaque psoriasis, this pooled estimate should be interpreted with the same population caveat noted in the Characteristics of included studies section.

Local skin adverse events. Three RCTs [9,24,28] provided comparable event counts for local skin reactions. Combination therapy was associated with a higher odds of local skin adverse events (OR = 2.35; 95% CI, 1.05 to 5.26; p=0.04), with low-to-moderate heterogeneity (I² = 38%; p=0.20) (Figure 4).

**Figure 4:**
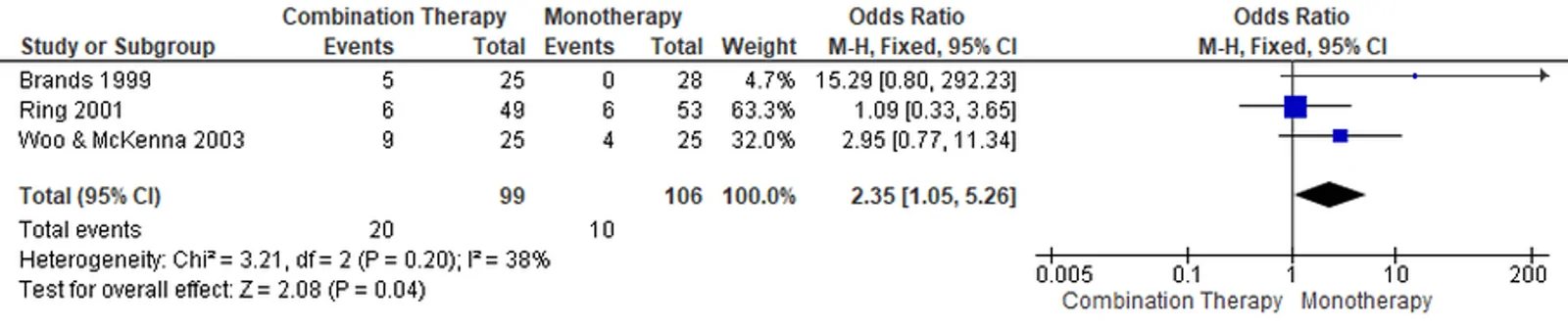
Forest plot of local skin adverse events Three RCTs [9,24,28] were included because they provided comparable group-level event counts for local skin reactions. Blue squares represent the effect estimate from each individual study; larger squares indicate greater study weight, and horizontal lines represent 95% confidence intervals. The black diamond represents the pooled effect estimate, with its width representing the pooled 95% confidence interval.

This estimate is derived from only three trials involving different topical agents, was assessed at the treatment-group level, and should therefore be interpreted cautiously and not generalized to all topical-phototherapy combinations.

Risk of Bias

Risk of bias was assessed in all 15 included RCTs using the Cochrane RoB 2 framework [18]. Most studies had at least some concerns, largely reflecting incomplete reporting of randomization procedures, lack of blinding, or potential deviations from intended interventions. Morison et al. [7] and Özkan et al. [19] were judged to have high overall risk of bias (Figure 5).

**Figure 5:**
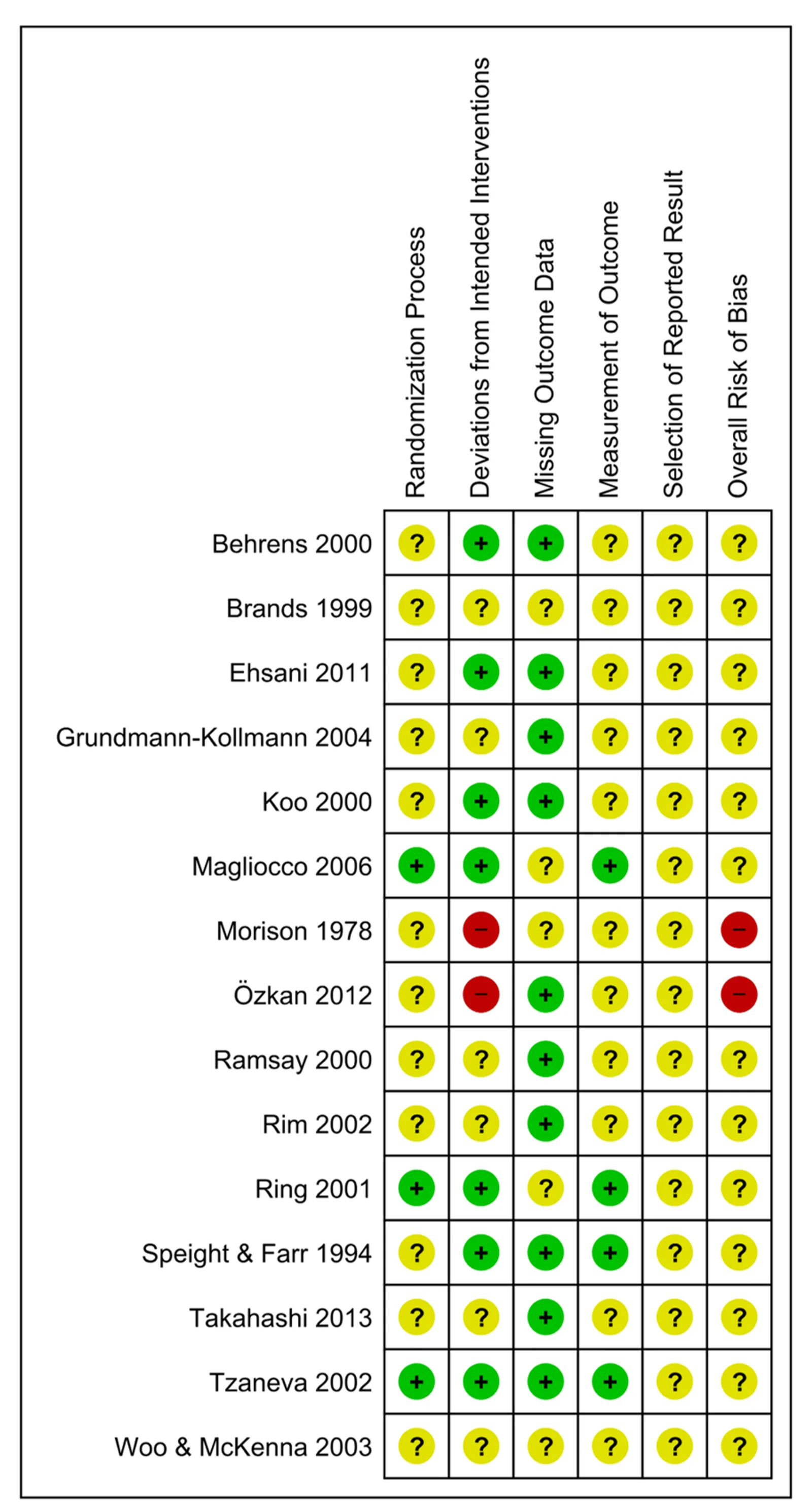
Cochrane RoB2 assessment for the included RCTs Risk of bias assessments are shown for the included RCTs [7-11,19-28]. Green indicates low risk of bias, yellow indicates some concerns, and red indicates high risk of bias across the RoB 2 domains and overall judgment.

Discussion

This systematic review and meta-analysis evaluated evidence from 15 RCTs comparing combination therapy with phototherapy alone for plaque psoriasis. Although the pooled analysis did not demonstrate a statistically significant improvement in PASI scores (SMD=0.26, 95% CI: −0.31 to 0.84; p=0.37), individual studies reported faster clinical improvement, earlier lesion clearance, and lower cumulative ultraviolet exposure with combination therapy [9,10,23-26]. Overall, the available evidence suggests that adjunctive topical therapy may improve treatment efficiency while maintaining comparable overall tolerability.

The observed findings are biologically plausible. Topical agents primarily reduce keratinocyte proliferation and local inflammation, whereas phototherapy exerts broader immunomodulatory and antiproliferative effects. Together, these complementary mechanisms may accelerate lesion clearance while reducing the cumulative ultraviolet dose required to achieve clinical improvement, potentially reducing long-term risks associated with cumulative ultraviolet exposure [24].

The pooled ORs for withdrawals due to adverse events was 0.98 (95% CI, 0.32-2.98), indicating comparable overall tolerability between treatment groups. In contrast, local skin reactions, particularly erythema, burning, and pruritus, were more frequent with combination therapy (OR = 2.35; 95% CI, 1.05-5.26). These reactions were generally mild to moderate and were reported particularly with retinoid-containing regimens [21,25,26].

Although the pooled PASI improvement did not reach statistical significance, individual studies demonstrated faster clinical improvement and reduced cumulative UV exposure with combination therapy. These observations of faster clinical improvement and lower cumulative UV exposure were reported in individual trials but were not consistently demonstrated across the evidence base or formally pooled, and should therefore be regarded as potential treatment-efficiency signals rather than established effects of combination therapy as a whole. The lack of statistical significance in the pooled analysis may be attributed to the limited statistical power and heterogeneity among the included studies. The observed heterogeneity may be explained by differences in phototherapy modalities (NB-UVB, BB-UVB, and PUVA), topical agents, treatment schedules, disease severity, follow-up duration, and outcome assessment methods. Notably, although included trials were required to meet our predefined plaque-type eligibility criteria, some enrolled patients with more localized or stable disease rather than moderate-to-severe involvement, and one trial (Woo and McKenna [9]) also included a subset of patients with guttate psoriasis, as noted above; this population heterogeneity is an important limitation, and our conclusions have been framed cautiously to avoid overstating the applicability of the findings specifically to moderate-to-severe plaque psoriasis.

In addition, several studies had small sample sizes and short follow-up periods, limiting the ability to evaluate long-term outcomes and relapse rates [9,10,23-26]. Subgroup analyses, sensitivity analyses, and formal assessment of publication bias, including funnel plot analysis, were not performed because the limited number of eligible studies and available data were insufficient to support reliable analyses. Furthermore, the inclusion of older RCTs introduced methodological variability, particularly in randomization and blinding, which may limit the applicability of these findings to current clinical practice. Additionally, PASI was not a routinely used outcome measure in phototherapy trials conducted during the earlier part of the study period, and because topical agents primarily act on localized lesions, they may improve local disease activity without producing a proportionate change in whole-body PASI scores; this measurement consideration should be borne in mind when interpreting the non-significant pooled difference between combination therapy and monotherapy.

Despite these limitations, the findings of this review have potential clinical relevance. Combination therapy may be considered as an adjunct to phototherapy for selected patients with plaque psoriasis, particularly when systemic therapies are unsuitable or contraindicated. This approach may help reduce cumulative ultraviolet exposure and shorten treatment duration while maintaining an acceptable safety profile. These findings may assist clinicians in selecting patients who may benefit from adjunctive topical therapy while balancing treatment efficacy, tolerability, and cumulative ultraviolet exposure. This approach is consistent with current international guidance: the joint American Academy of Dermatology - National Psoriasis Foundation (AAD-NPF) phototherapy guideline supports combining phototherapy with topical agents to improve response and reduce cumulative ultraviolet exposure [6], while the EuroGuiDerm systemic treatment guideline for psoriasis vulgaris reflects the expanding role of biologic and other targeted systemic therapies in contemporary moderate-to-severe psoriasis management [29], a context that should be considered when weighing the place of phototherapy-based combination regimens, largely evaluated in older trials, within current treatment pathways.

Future research should focus on large and multicenter RCTs with standardized PASI endpoints and prolonged follow-up to evaluate response durability. Future studies should also incorporate patient-reported outcomes, including the Dermatology Life Quality Index (DLQI) and treatment satisfaction, to better evaluate the patient-centered impact of combination therapy. In addition, future mechanistic studies incorporating advanced imaging techniques and molecular biomarkers may help clarify the biological basis of the differential clinical response and local adverse-event profiles observed with combination therapy.

In summary, combination therapy did not demonstrate a statistically significant improvement in pooled efficacy, but was associated with faster lesion clearance, earlier clinical improvement, and an acceptable safety profile. These findings support the use of combination therapy as a reasonable adjunct to phototherapy in appropriately selected patients with plaque psoriasis.

## Conclusions

The pooled analysis showed no statistically significant difference in PASI improvement between combination therapy and phototherapy alone. However, individual studies suggested faster lesion clearance and reduced cumulative UV exposure with specific combination regimens, although these effects were not consistently demonstrated across the pooled evidence. Safety outcomes were generally comparable between treatment groups, although local skin reactions were more frequent with combination therapy and were typically mild and manageable. In addition, some included trials enrolled patients with more localized or stable disease rather than moderate-to-severe involvement, so these findings should not be generalized beyond the populations studied, and their applicability specifically to moderate-to-severe plaque psoriasis should be interpreted with caution. Taken together, the findings do not establish a consistent overall efficacy advantage of combination therapy over phototherapy alone, although some specific topical-phototherapy combinations may accelerate clinical response or reduce treatment burden in selected patients with plaque psoriasis when used as an adjunct to phototherapy. Further large, well-designed RCTs with standardized treatment protocols, longer follow-up, and patient-reported outcome measures are needed to strengthen the current evidence.

## Appendices

Appendix A

**Table 3:** Prior treatment and washout criteria for included studies

First Author (Year)	Previous Treatment / Washout
Takahashi et al., 2013 [11]	Washout of 2 weeks for topical therapies and 4 weeks for systemic treatment.
Özkan et al., 2012 [17]	Not reported.
Ehsani et al., 2011 [20]	Not reported.
Magliocco et al., 2006 [21]	Previous treatments failed prior to enrollment. Washout of 1 month for systemic therapies, PUVA, UVB, topical therapies, and other retinoids.
Grundmann-Kollmann et al., 2004 [22]	Prior use of glucocorticoids, calcipotriol, dithranol, tazarotene, or UVB.
Woo and McKenna, 2003 [9]	No phototherapy or systemic antipsoriatic treatment within 2 months.
Rim et al., 2002 [23]	Not reported; 4-week washout for systemic/UV therapy.
Tzaneva et al., 2002 [10]	All phototherapy, systemic, and topical psoriasis treatments discontinued for 4 weeks before the study. Only non-medicated emollients were permitted during the study; no other psoriasis treatments were allowed.
Ring et al., 2001 [24]	Washout of 2 months for systemic/intralesional therapy or photo(chemo)therapy.
Behrens et al., 2000 [25]	Not reported.
Koo et al., 2000 [26]	Not reported.
Ramsay et al., 2000 [8]	Washout of 2 months for systemic therapy or phototherapy.
Speight and Farr, 1994 [27]	No topical treatment for 2 weeks prior to enrollment; 6 of 13 patients had previously used calcipotriol with little or no effect.
Brands et al., 1999 [28]	No phototoxic drugs; no recent phototherapy.
Morison et al., 1978 [7]	Not reported.

Appendix B: Complete search terms by database

PubMed/MEDLINE: (psoriasis[MeSH Terms] OR plaque psoriasis[Title/Abstract]) AND (phototherapy[MeSH Terms] OR ultraviolet therapy[Title/Abstract] OR PUVA[Title/Abstract] OR UVB[Title/Abstract]) AND (topical[Title/Abstract] OR corticosteroids[Title/Abstract] OR vitamin D analogs[Title/Abstract] OR calcipotriol[Title/Abstract] OR tazarotene[Title/Abstract]) AND (randomized controlled trial[Publication Type]OR randomized*[Title/Abstract])

Embase: (psoriasis OR plaque psoriasis) AND (phototherapy OR ultraviolet therapy OR PUVA OR UVB) AND (topical OR corticosteroids OR vitamin D analogs OR calcipotriol OR tazarotene) AND (randomized controlled trial OR randomized)

Scopus: (psoriasis OR plaque psoriasis) AND (phototherapy OR ultraviolet therapy OR PUVA OR UVB) AND (topical OR corticosteroids OR vitamin D analogs OR calcipotriol OR tazarotene) AND (randomized controlled trial OR randomized)

ScienceDirect: (psoriasis OR plaque psoriasis) AND (phototherapy OR ultraviolet therapy OR PUVA OR UVB) AND (topical OR corticosteroids OR vitamin D analogs OR calcipotriol OR tazarotene) AND (randomized controlled trial OR randomized)

Cochrane CENTRAL: (psoriasis OR plaque psoriasis) AND (phototherapy OR ultraviolet therapy OR PUVA OR UVB) AND (topical OR corticosteroids OR vitamin D analogs OR calcipotriol OR tazarotene) AND (randomized controlled trial OR randomized)

Web of Science: (psoriasis OR plaque psoriasis) AND (phototherapy OR ultraviolet therapy OR PUVA OR UVB) AND (topical OR corticosteroids OR vitamin D analogs OR calcipotriol OR tazarotene) AND (randomized controlled trial OR randomized)

ClinicalTrials.gov: (psoriasis OR plaque psoriasis) AND (phototherapy OR ultraviolet therapy OR PUVA OR UVB) AND (topical OR corticosteroids OR vitamin D analogs OR calcipotriol OR tazarotene) AND (randomized controlled trial OR randomized)

## References

[1] Griffiths CE, Armstrong AW, Gudjonsson JE, Barker JN (2021). Psoriasis. Lancet.

[2] Sieminska I, Pieniawska M, Grzywa TM (2024). The immunology of psoriasis-current concepts in pathogenesis. Clin Rev Allergy Immunol.

[3] Kemény L, Varga E, Novak Z (2019). Advances in phototherapy for psoriasis and atopic dermatitis. Expert Rev Clin Immunol.

[4] Elmets CA, Korman NJ, Prater EF (2021). Joint AAD-NPF Guidelines of care for the management and treatment of psoriasis with topical therapy and alternative medicine modalities for psoriasis severity measures. J Am Acad Dermatol.

[5] Thompson KG, Kim N (2020). Distinguishing myth from fact: photocarcinogenesis and phototherapy. Dermatol Clin.

[6] Elmets CA, Lim HW, Stoff B (2019). Joint American Academy of Dermatology-National Psoriasis Foundation guidelines of care for the management and treatment of psoriasis with phototherapy. J Am Acad Dermatol.

[7] Morison WL, Parrish JA, Fitzpatrick TB (1978). Controlled study of PUVA and adjunctive topical therapy in the management of psoriasis. Br J Dermatol.

[8] Ramsay CA, Schwartz BE, Lowson D, Papp K, Bolduc A, Gilbert M (2000). Calcipotriol cream combined with twice weekly broad-band UVB phototherapy: a safe, effective and UVB-sparing antipsoriatric combination treatment. The Canadian Calcipotriol and UVB study group. Dermatology.

[9] Woo WK, McKenna KE (2003). Combination TL01 ultraviolet B phototherapy and topical calcipotriol for psoriasis: a prospective randomized placebo-controlled clinical trial. Br J Dermatol.

[10] Tzaneva S, Hönigsmann H, Tanew A, Seeber A (2002). A comparison of psoralen plus ultraviolet A (PUVA) monotherapy, tacalcitol plus PUVA and tazarotene plus PUVA in patients with chronic plaque-type psoriasis. Br J Dermatol.

[11] Takahashi H, Tsuji H, Ishida-Yamamoto A, Iizuka H (2013). Comparison of clinical effects of psoriasis treatment regimens among calcipotriol alone, narrowband ultraviolet B phototherapy alone, combination of calcipotriol and narrowband ultraviolet B phototherapy once a week, and combination of calcipotriol and narrowband ultraviolet B phototherapy more than twice a week. J Dermatol.

[12] Page MJ, McKenzie JE, Bossuyt PM (2021). The PRISMA 2020 statement: an updated guideline for reporting systematic reviews. BMJ.

[13] Fredriksson T, Pettersson U (1978). Severe psoriasis-oral therapy with a new retinoid. Dermatologica.

[14] Langley RG, Ellis CN (2004). Evaluating psoriasis with Psoriasis Area and Severity Index, Psoriasis Global Assessment, and Lattice System Physician's Global Assessment. J Am Acad Dermatol.

[15] Ouzzani M, Hammady H, Fedorowicz Z, Elmagarmid A (2016). Rayyan-a web and mobile app for systematic reviews. Syst Rev.

[16] (2026). Cochrane handbook for systematic reviews of interventions. https://training.cochrane.org/handbook/archive/v6.4.

[17] (2026). Review Manager (RevMan). https://revman.cochrane.org/.

[18] Sterne JA, Savović J, Page MJ (2019). RoB 2: a revised tool for assessing risk of bias in randomised trials. BMJ.

[19] Ozkan I, Köse O, Ozmen I, Arca E (2012). Efficacy and safety of non-laser, targeted UVB phototherapy alone and in combination with psoralen gel or calcipotriol ointment in the treatment of localized, chronic, plaque-type psoriasis. Int J Dermatol.

[20] Ehsani AH, Ghaninejad H, Kiani A, Daneshpazhooh M, Hosseini SH, Noormohammadpoor P (2011). Comparison of topical 8-methoxypsoralen and narrowband ultraviolet B with narrowband ultraviolet B alone in treatment-resistant sites in plaque-type psoriasis: a placebo-controlled study. Photodermatol Photoimmunol Photomed.

[21] Magliocco MA, Pandya K, Dombrovskiy V, Christiansen L, Wong Y, Gottlieb AB (2006). A randomized, double-blind, vehicle-controlled, bilateral comparison trial of bexarotene gel 1% versus vehicle gel in combination with narrowband UVB phototherapy for moderate to severe psoriasis vulgaris. J Am Acad Dermatol.

[22] Grundmann-Kollmann M, Ludwig R, Zollner TM (2004). Narrowband UVB and cream psoralen-UVA combination therapy for plaque-type psoriasis. J Am Acad Dermatol.

[23] Rim JH, Choe YB, Youn JI (2002). Positive effect of using calcipotriol ointment with narrow-band ultraviolet B phototherapy in psoriatic patients. Photodermatol Photoimmunol Photomed.

[24] Ring J, Kowalzick L, Christophers E (2001). Calcitriol 3 microg g-1 ointment in combination with ultraviolet B phototherapy for the treatment of plaque psoriasis: results of a comparative study. Br J Dermatol.

[25] Behrens S, Grundmann-Kollmann M, Schiener R, Peter RU, Kerscher M (2000). Combination phototherapy of psoriasis with narrow-band UVB irradiation and topical tazarotene gel. J Am Acad Dermatol.

[26] Koo JY, Lowe NJ, Lew-Kaya DA, Vasilopoulos AI, Lue JC, Sefton J, Gibson JR (2000). Tazarotene plus UVB phototherapy in the treatment of psoriasis. J Am Acad Dermatol.

[27] Speight EL, Farr PM (1994). Calcipotriol improves the response of psoriasis to PUVA. Br J Dermatol.

[28] Brands S, Brakman M, Bos JD, de Rie MA (1999). No additional effect of calcipotriol ointment on low-dose narrow-band UVB phototherapy in psoriasis. J Am Acad Dermatol.

[29] Nast A, Smith C, Spuls PI (2020). EuroGuiDerm guideline on the systemic treatment of psoriasis vulgaris - part 1: treatment and monitoring recommendations. J Eur Acad Dermatol Venereol.

